# Standard ophthalmology residency training in China: an evaluation of resident satisfaction on training program in Guangdong Province

**DOI:** 10.1186/s12909-023-04527-3

**Published:** 2023-08-03

**Authors:** Xiaonan Yang, Danying Zheng, Pengxia Wan, Xiaoling Luo, Mingzhi Zhang, Liang Zhang, Shaochong Zhang, Jingjing Huang, Yehong Zhuo

**Affiliations:** 1 Zhongshan Ophthalmic Center, State Key Laboratory of Ophthalmology, Sun Yat-sen University, No.7, Jinsui Road, Guangzhou, China; 2 Department of Ophthalmology, the First Affiliated Hospital, Sun Yat-sen University, Guangzhou, 510080 Guangdong Province China; 3 Department of Ophthalmology, Shenzhen People’s Hospital Second Clinical Medical College of Jinan University, Shenzhen, China; 4 Joint Shantou International Eye Center, Shantou University & the Chinese University of Hong Kong, Shantou, 515041 China; 5 Department of Ophthalmology, Guangdong Provincial People’s Hospital, Guangdong Academy of Medical Sciences, No. 106, Zhongshan Er Road, Yuexiu District, Guangzhou, Guangdong China; 6 Shenzhen Eye Hospital affiliated to Jinan University, Shenzhen, China

**Keywords:** Ophthalmology training, Performance, Residents, Standardized residency training

## Abstract

**Background:**

National standardized training for resident doctors (STRD) in mainland China has been formally established since 2014 as a kind of postgraduate education. The purpose of this survey was to assess the satisfaction of the training residents in Guangdong Province on the ophthalmology STRD program after a duration of 5 years.

**Method:**

A 48-item survey was sent to all postgraduate ophthalmology residents from bases in Guangdong Province to inquire about their attitude towards the program. The survey contained questions about demographic and work-related information, job satisfaction, psychological resilience, and job performance. All responses were verified, and invalid questionnaires were excluded. Statistical analyses were performed using SPSS software version 22.0 (SPSS, Inc., Chicago, IL). Multiple logistic regression analysis was used to evaluate the factors (demographic information, working environment, clinical exposure, supervision and hands-on training opportunities, and involvement in academic activities) impacting the overall satisfaction. P < 0.05 was considered statistically significant.

**Results:**

A total of 471/635 (74.17%) valid questionnaires were returned from all the STRD bases of Guangdong Province, which included 38 hospitals. 60.3% of the respondents reported overall satisfaction with their training. The satisfaction with operative teaching (60.7%) was slightly lower than the other settings of teaching experience (above 65%). Meanwhile, the satisfaction on different secessions of operative experience was all below 70%, of which in the areas of cornea and orbit were 55.42% and 57.53%, respectively. Some potential factors were found to affect general satisfaction, including the training grade, marriage, working time, income level, the doctor-patient relationship, family members working as doctors, the time proportion spent on writing medical documents during clinical work, and the frequency of attending academic meetings. Improvement was observed in both performing and reporting clinical examinations in the last year of training in comparison to the first year. Finally, 82.8% of the residents acknowledged this training was helpful for future clinical work. The first five career preferences for residents were cataract (67.1%), refractive surgery (42.3%), vitreo-retina (36.5%), optometry (28.7%), and oculoplastic (27.2%).

**Conclusion:**

Ophthalmology residents in Guangdong Province expressed comparable satisfaction with the STRD program. To further improve satisfaction, factors such as resident subsidy, harmonious marriage, the patient-doctor relationship, and chances of attending academic conferences should be emphasized.

**Supplementary Information:**

The online version contains supplementary material available at 10.1186/s12909-023-04527-3.

## Background

Under the guidance of the National Health and Family Planning Commission in mainland China, the establishment of the national standardized training for resident doctors (STRD) has served as a kind of postgraduate education to improve the quality of physician training since 2014 [1, 2]. Even though the diverse subspecialties within the program ensure all trainees can become safe and independent junior practitioners with sophisticated job performance, standardized residency training is relatively new in China, especially in ophthalmologists’ training [3].

The introduction of STRD in Ophthalmology is crucial because it prepares graduates for the rapidly evolving field dominated by technological advances, procedural diversification, and practical skills. Theoretically, ophthalmology STRD in China could start immediately after the 5-year medical school training, which consisted of basic and clinical science courses in the early years and followed by clerkship and internship training in the last 2 years. Students may start their residency training with the possibility of pursuing a Master’s (usually 3 years) or Doctor of Philosophy degree (usually 3 + 3 years or more) in clinical medicine. Specifically, some graduates could apply for master’s degrees and STRD concurrently, which is named the professional master. Usually, the STRD for ophthalmologists in China has a duration of three years for all trainees regardless of their degree, while it varies from 2 to 7 years internationally.

Ophthalmology residents under STRD in China were given annual assessments on clinical theory and practice. However, their satisfaction with the program was left unmeasured. To fill the void of such measurement, this research aimed to provide a comprehensive evaluation of the ophthalmology residency programs in Guangdong Province through anonymous and independent responses from ophthalmology residents.

## Methods

A cross-sectional survey was conducted from September 2019 to December 2019 in all the training bases of 38 hospitals in Guangdong Province. The ophthalmology residents, including postgraduate years (PGY) 1, 2, and 3, were invited to participate in this survey. Ethical approval was taken from all the Ethics Committee of 38 ophthalmology bases **(shown in the ethics approval and consent to participate section of the Declarations part in detail)** of Guangdong province before September 2019. This study was conducted according to the principles of the Declaration of Helsinki.

Questionnaires were sent via mobile application (Wechat®) to all resident ophthalmologists, with a cover letter explaining the purpose of the questionnaire. The responsible person in the administrative office at each hospital was contacted to ensure that all ophthalmology residents had received the questionnaires.

The questionnaire was self-administered, requiring approximately 20 min to complete. Respondents were instructed to complete the questionnaire independently. Data were returned to the coordinating center at Zhongshan Ophthalmic Center and were extracted for further analysis.

Potential participants were told that returning the form was indicative of informed consent, and anonymity could be maintained without the need for a signature.

### Questionnaire

The questionnaire was designed based on published literature regarding Ophthalmology resident training [4–6]. This questionnaire in Chinese was designed to obtain information from resident trainees on the following 5 categories: (1) Demographic information (gender, age, educational level, and years of training); (2) Working environment; (3) Clinical exposure, supervision, and hands-on training opportunities; (4) Involvement in academic and research activities; (5) Satisfaction.

Drafted questionnaires were administered to 20 ophthalmology residents in a pilot survey before initiation. After modification, the final version with 48 items in 5 parts was confirmed (English and Chinese version of the questionnaire was available as supplementary material online).

### Statistical analyses

All responses were verified and invalid questionnaires were excluded. Invalid questionnaires were defined as (1) unclear and incomplete responses, (2) more than one response to each one-answer question, and (3) duplicate questionnaires. Statistical analyses were performed using SPSS software version 22.0 (SPSS, Inc., Chicago, IL). Multiple logistic regression analysis was used to evaluate the factors (demographic information, working environment, clinical exposure, supervision and hands-on training opportunities, and involvement in academic and research activities) impacting the overall satisfaction. P < 0.05 was considered statistically significant.

## Results

During the period of this evaluation, there were a total of 635 post-graduate ophthalmology residents in Guangdong Province, China. A total of 471 valid questionnaires were returned from 38 training bases of Guangdong Province. The response rate was 74.17%. The demographic characteristics of the involved residents are shown in Table 1. In this survey, the STRD program included both theoretical and operation teaching.

**Table 1:** Demographic characteristics of all respondents (n = 471)

Characteristic	n (%)
**Age**	
21–25	227 (48.2)
26–30	217 (46.1)
> 31	27 (5.7)
**Gender distribution**	
Female	326 (69.2)
Male	145 (30.8)
**Level of training**	
PGY-1	182 (38.6)
PGY-2	159 (33.8)
PGY-3	130 (27.6)
**Education**	
Bachelor	232 (49.3)
Master	193 (41.0)
Doctor	46 (10.0)
**Marital status**	
Married	14.4%
Single	85.6%

PGY means postgraduate years.

### General satisfaction

**Fig. 1:**
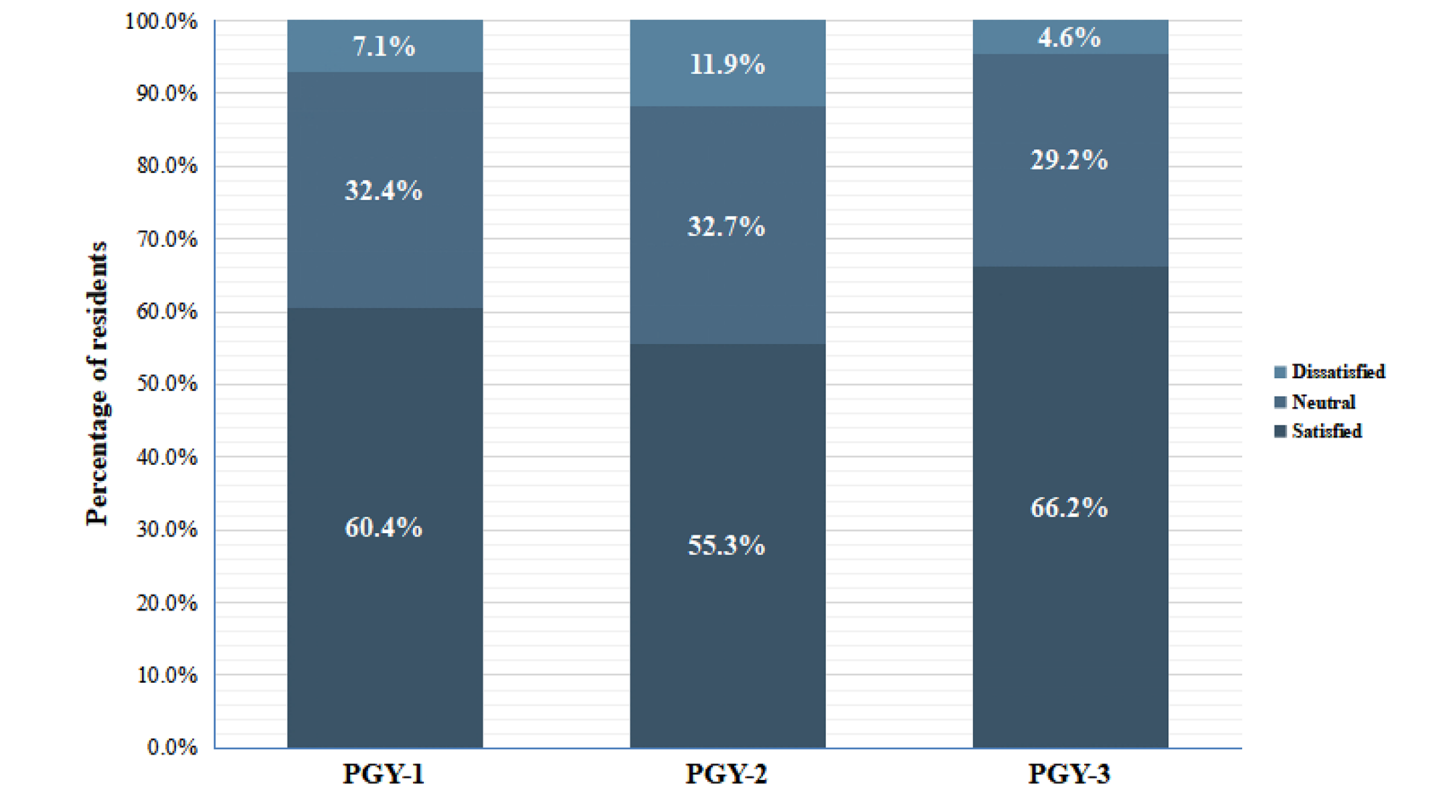
The overall satisfaction evaluation from residents at different lever of training

The overall satisfaction of residents with their residency training is shown in Table 2. 60.3% of the respondents reported overall satisfaction with their training. 60.4% of the PGY-1 residents, 55.3% of the PGY-2 residents and 66.2% of the PGY-3 residents felt satisfied with the training (Fig. 1). In addition, 82.8% of the residents acknowledged this training was helpful for future clinical work.

**Table 2:** Overview of residency training satisfaction (n = 471)

Questions	Satisfied (%)		Neutral (%)	Dissatisfied (%)
What is your overall level of satisfaction you’re your ophthalmology residency program?	60.3		31.63	8.07
Is the program helpful for the future clinical work?	82.8		15.50	1.70
How do you feel about the quality of teaching in the following settings?				
Pre-employment training	76.01		19.32	4.67
Clinic/outpatient office Hospital-based rounds	65.18		28.45	6.37
Operating teaching	60.72		29.94	9.34
Case discussion	75.58		22.08	2.34
Teaching grand rounds	75.16		21.66	2.97
Hospital-based academic activities	75.37		22.08	2.55
Academic conferences	73.25		23.99	2.76
How do you feel about the operative experience in the following areas?				
Case variety	67.72		26.75	5.52
Case complexity	68.58		27.39	4.03
Case volume	69.64		22.72	7.64
Cataract	66.45		26.11	7.43
Glaucoma	60.51		32.48	7.01
Cornea	55.42		36.31	8.28
Retina	64.33		28.87	6.79
Strabismus	62.21		31.85	5.94
Orbital	57.53		35.03	7.43

The potential factors affecting general satisfaction are shown in Table 3. It was shown that PGY-3 residents had the highest satisfaction (odds ratio [OR] of PGY-1 = 1.99 (p = 0.023), OR PGY-2 = 2.66 (p = 0.001)). Married residents were more satisfied with this training than single residents (OR = 0.43, p = 0.031). In addition, compared to the professional master students who received a monthly aid of about 1000 Chinese yuan, the practitioners who received more than 4000 Chinese yuan monthly reported higher satisfaction scores. Furthermore, a satisfaction score above 90 on the doctor-patient relationship had a positive effect on overall STRD satisfaction. Residents with no family members in the medical field tended to have lower overall satisfaction (OR = 2.60, p = 0.019). The less time spent on writing medical documents compared to clinical work had a statistically significant effect on overall satisfaction. Specifically, residents spending more than 80% of their working time on writing medical documents had the lowest overall satisfaction with the STRD program. For the frequency of attending academic meetings per year, only the group ‘zero’ was found to be different from the reference group ‘>5’ in affecting overall STRD satisfaction.

**Table 3:** Summary of ordinal regression analysis with overall satisfaction as the dependent variable

Item	β	p value	Odds ratio	95% CI	
				Lower limits	Upper limits
**Gender**					
Male	0.099	0.683	1.10	-0.375	0.573
Female			Ref		
**Age**					
21–25	-0.162	0.809	0.85	-1.482	1.157
26–30	0.365	0.556	1.44	-0.851	1.581
> 30			Ref		
**Level of training**					
2019	0.688	***0.023***	1.99	0.095	1.281
2018	0.979	***0.001***	2.66	0.396	1.563
2017			Ref		
**Education background**					
undergraduate	0.336	0.579	1.40	-0.851	1.523
master	0.11	0.844	1.112	-0.989	1.210
PhD			Ref		
**Marital status**					
Married	-0.852	***0.031***	0.43	-1.583	-0.121
Single			Ref		
**How many days need to spend on working?**					
< 6 days	0.054	0.906	1.06	-0.843	0.951
6 days	-0.301	0.340	0.74	-0.918	0.316
7 days	-0.093	0.761	0.91	-0.695	0.508
As appropriate			Ref		
**The most time-consuming tasks as an ophthalmology resident**					
Out-patient clinic	-0.434	0.6	0.65	-2.056	1.189
Surgery or operations	0.092	0.912	1.10	-1.528	1.711
Medical records	-0.553	0.468	0.58	-2.048	0.941
doctor-patient communication			Ref		
**The time proportion writing medical documents spent in clinical work**					
< 20%	-1.88	***0.035***	0.15	-3.632	-0.128
20–40%	-1.907	***< 0.001***	0.15	-2.893	-0.921
40–60%	-1.118	***0.009***	0.33	-1.956	-0.279
60–80%	-0.611	0.17	0.54	-1.483	0.261
> 80%			Ref		
**The time scientific research spent every week**					
zero	0.139	0.779	1.15	-0.835	1.113
1–5 h	0.087	0.784	1.09	-0.535	0.709
5–10 h	0.176	0.602	1.19	-0.486	0.838
Over 10 h			Ref		
**Types of research participation**					
Basic research	0.255	0.641	1.29	-0.817	1.328
Clinical research	-0.209	0.681	0.81	-1.206	0.788
Both	-0.018	0.973	0.98	-1.052	1.017
Neither			Ref		
**Monthly income**					
3000–4000	-0.528	0.104	0.59	-1.165	0.108
4001–5000	-1.022	***0.030***	0.36	-1.943	-0.1
5001–6000	-1.561	***0.001***	0.21	-2.464	-0.658
6001–7000	-1.3	***0.010***	0.27	-2.287	-0.313
> 7000	-0.705	0.145	0.49	-1.653	0.243
Month-aid for graduate			Ref		
**Is the income worthy of work**					
Yes	-0.811	***0.013***	0.44	-1.451	-0.172
No			Ref		
**In your opinion, is your current income commensurate with your initial investment in education?**					
Income exceeds initial investment	Not available				
Income and initial investment are basically commensurate	-0.113	0.739	0.89	-0.777	0.551
The income is lower than the upfront investment			Ref		
**Satisfaction score of the doctor-patient relationship in the current hospital**					
< 59	3.004	***< 0.001***	20.17	1.571	4.436
60–80	1.653	***< 0.001***	5.22	1.049	2.258
80–90	0.658	***0.019***	1.93	0.106	1.211
> 90			Ref		
**The number of family members practicing medicine?**					
None	0.955	***0.001***	2.60	0.374	1.535
One	0.449	0.204	1.57	-0.243	1.142
More than one			Ref		
**Frequency of attending academic meetings**					
Zero	1.547	***0.001***	4.70	0.619	2.474
1 or 2 times per year	0.479	0.090	1.61	-0.075	1.033
3 to 5 times per year	0.436	0.146	1.55	-0.152	1.023
> 5			Ref		

The P value of “Parallel line test” was 0.878. McFadden R^2^ = 0.223. The overall satisfaction in the ordinal regression analysis was divided into three levels, including satisfied, neutral, and dissatisfied.

In addition, factors like age, gender, highest educational degree, time used for work per day, the match degree between income and initial investment, time for clinical work per week, the largest component of one’s clinical work, time of attending continuing medical education lectures in the recent 6 months, time for research work per week, and the kinds of the research work, did not affect the overall satisfaction.

### Quality of teaching

**Fig. 2:**
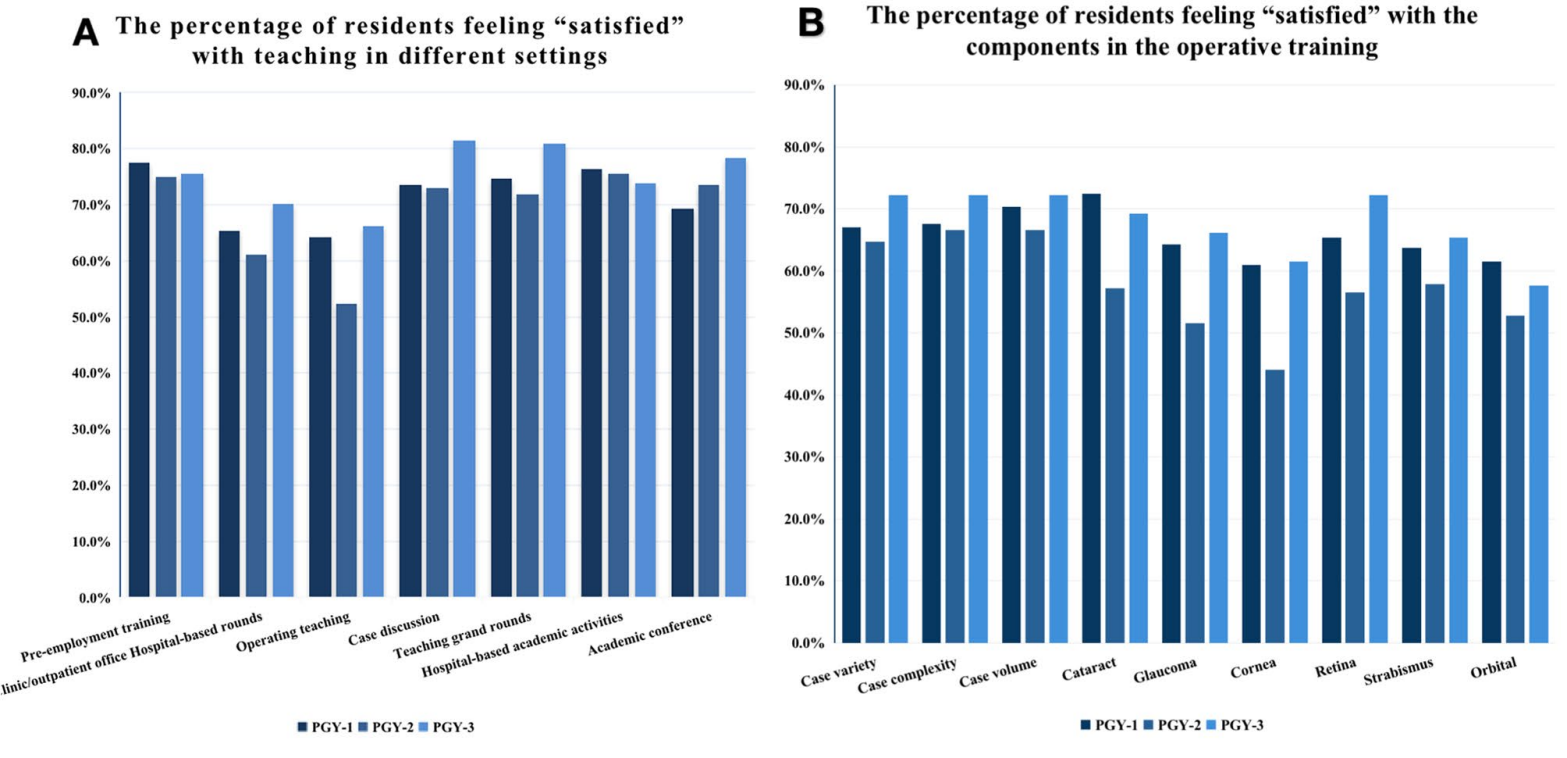
The distribution of residents who were satisfied with the components in the teaching (**A**) or the operative training (**B**)

Most of the respondents were satisfied with the quality of teaching in different settings (Table 2). The satisfaction rates for operative training in the cornea and orbit departments were 55.42% and 57.53%, respectively (Table 2). However, only 52.2% of PYG-2 residents reported satisfaction with operative teaching (Fig. 2A). Figure 2B displays the distribution of residents who were satisfied with the various components of operative teaching, such as variety, complexity, and exposure to different subspecialties.

For residents, various streams could be employed to boost additional knowledge/training on clinical knowledge, such as reading journals/books/compact disks (36.90%), contacting residency mentors for advice (17.98%), attending seminars and meetings (12.46%), communicating with other residents (12.46%), and participating in continuing education courses (11.76%).

Contrary to the wide availability of different learning methods, outstanding academic performance was achieved by a few residents. In detail, only 17.62% and 22.29% of the respondents have published academic articles on Chinese and international medical journal, respectively. Regarding the reasons for publishing articles or attending conferences, 40.76% of residents stated that it helps them obtain medical degrees. 23.14% of them used it as a platform to solve clinical problems, while 17.62% found it to be an interesting pursuit. 10.62% cited career promotion pressure as their motivation, and 7.86% recommended it as a means to enhance their academic status.

Furthermore, with regards to the influence of participating in academic activities during clinical work, 63.48% of respondents believed it to be beneficial in enhancing clinical thinking, while 29.72% felt that it only leads to fatigue and is a waste of time. The remaining 6.79% reported no impact on their work.

### Operative experience

The satisfaction with the operative experience was slightly lower than that of the teaching experience (Table 2). Most of the residents were satisfied with the cases’ variety (67.72%), complexity (68.58%), and volume (69.64%). Only 44% of the PGY-2 residents were satisfied with their corneal operative experience.

**Fig. 3:**
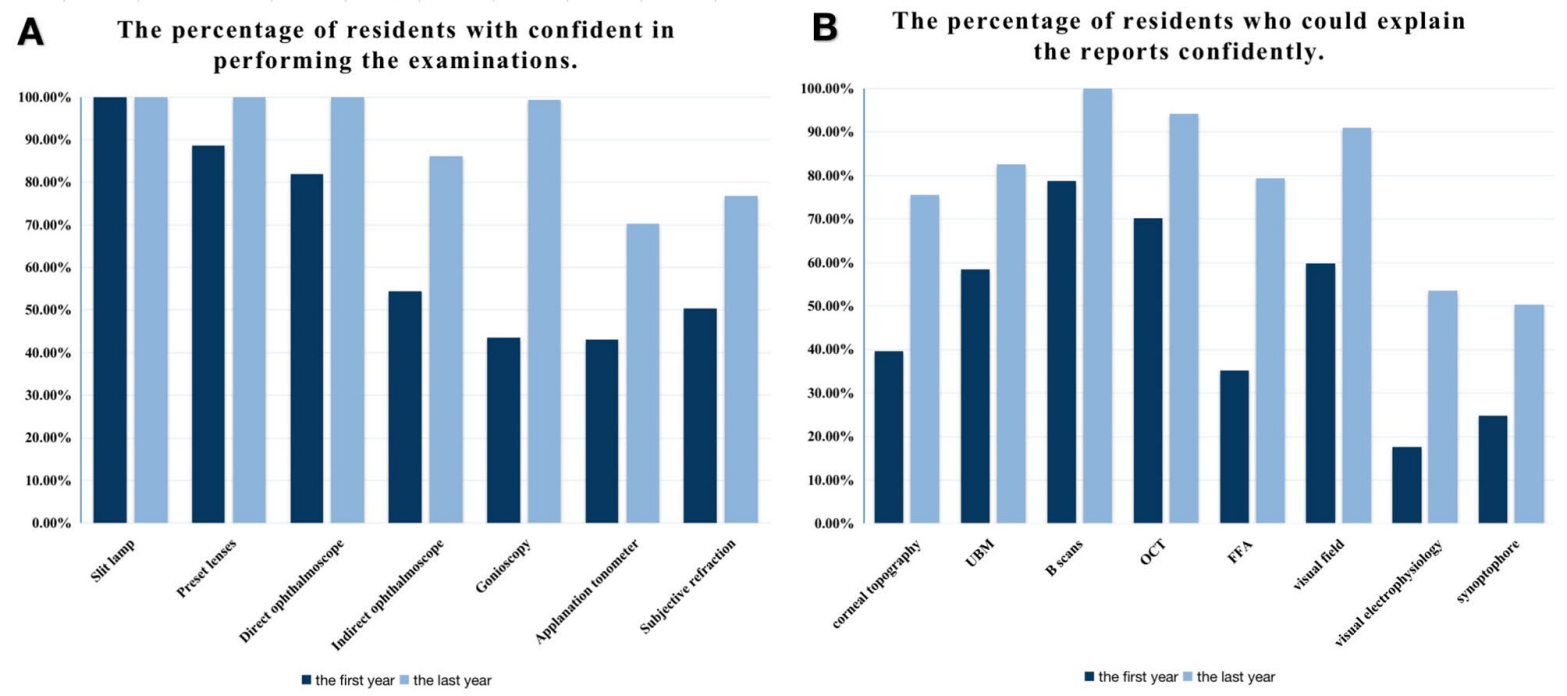
The percentages of the residents who acquired the examination skills (**A**), and who had the ability to analyze the examination reports (**B**)

The percentages of the residents who had confidence in acquiring examination skills and analyzing examination reports in the first year and after the training are shown in Fig. 3. In the last year, significant improvements were observed in both the examination skills and the ability to analyze examination reports compared with those in the first year.

Additionally, all residents reported that they could handle subconjunctival injection, retrobulbar injection, anterior chamber paracentesis, and intravitreal injection confidently when the residency training was completed. Meanwhile, 81.29%, 79.35%, 66.45%, and 9.68% of the residents could complete chalazion excision, sutures of the eyelid injury, corneoscleral suture, and phacoemulsification, respectively. Only 6.45% of them could not complete the operations above.

### Career preferences

The career preference of the involved residents is shown in Table 4.

67.1% of residents chose cataract as their first choice, while only 1.1% chose genetic disease as the first choice. The main factor influencing the choice of specialized subject was interest (selected by 84.50%), while occupational accomplishment (selected by 46.92%), salary (selected by 34.82%) and flexibility (selected by 25.27%) were also important. Some residents also chose low technical difficulty (selected by 6.16%), and others (selected by 4.67%) as the reasons of their choices.

**Table 4:** Current career preference/aspiration

Currently, what is your career preference/aspiration?		
Answer options	Response percent	Response count
Cataract	67.1%	316
Refractive surgery	42.3%	199
Vitreo-retina	36.5%	172
Optometry	28.7%	135
Oculoplastics	27.2%	128
Medical retina	23.6%	111
Glaucoma	22.3%	105
Cornea	18.9%	89
Ocular traumas	10.4%	49
Paediatrics & strabismus	9.8%	46
Uveitis	5.9%	28
Orbit	4.5%	21
Genetic	1.1%	5
Other	1.9%	9

## Discussion

In China, continuous improvement of professional quality in practice training is diving in. Therefore, paying attention to trainees’ feedback could aid in the sustainable innovation of STRD. To bridge this gap, we conducted a survey among all ophthalmology training bases in Guangdong Province, consisting of 38 tertiary-class hospitals. To our knowledge, this is the investigation of maximum sample size (471 responses) with a high response rate of 74.17% [7], ensuring high validity for our study [8]. The overall satisfaction and all the related influencing factors were analyzed in this survey.

The involved residents included undergraduate (49.3%), graduate (41%) and doctoral (10%) students, and they all completed at least 5 years of a medical school curriculum. This is different from other countries such as the US, in which students must complete 4 years of a college curriculum, followed by medical school, and finally pass the United States Medical Licensing Examination (USMLE) examinations before being qualified for postgraduate ophthalmology training [9]. Interestingly, only 5.7% of the involved residents were older than 30 years of age, who were younger than the ones in other countries. There were fewer married residents in our survey (14.4%) compared to surveys in other countries. More surprisingly, the female/male ratio in this survey was 2.26 (326/145), while it was less than 1.0 internationally [4]. In fact, the physician workforce in China has been female predominant since 2005 [10, 11]. The potential explanation for this phenomenon was that there was no significant ongoing female gender bias in China [12]. Also, in China, ophthalmologists are paid less for clinical work, receive smaller research grants, have fewer professional ties with the medical industry, have fewer publications in peer-reviewed ophthalmology journals, and hold fewer journal editors’ chairs. Consequently, male medical students had less tendency to be ophthalmologists [10].

In summary, 60.3% of the Ophthalmology residents in Guangdong Province were satisfied with the STRD program, while 31.63% had a neutral attitude. 82.8% of the respondents thought this program was helpful for future clinical work. Notably, PGY-3 trainees were more satisfied with STRD than PGY-1 trainees (Table 3), which implied that the STRD program benefits the trainees as they continue their medical careers. In our survey, the percentage of overall satisfaction was higher in residents with doctoral (73.91%) degree than with bachelor (57.33%) or master (60.62%) degrees. It was possible that trainees with doctoral degrees had more opportunities to do independent operations, and thus reported a higher satisfaction score. Certainly, new innovations are still needed to not only improve the residents’ satisfaction but also improve their clinical competency. We found several factors that impacted the overall satisfaction (Table 2). Improving income, proper working time arrangement, a harmonious doctor-patient relationship, and chances for attending academic meetings, would boost satisfaction [13, 14]. However, compared with ophthalmology residents training program in the U.S. (93.6% responded that they were highly satisfied with their programs), the overall satisfaction is relatively low in our survey [6]. Insufficient opportunities, the limited availability of training positions, the lack of adequate teaching by attending physicians, and difficulty in securing a job after training, are some possible explanations for such low satisfaction, and deserve to be further emphasized in China [3].

In addition, less than 70% of the residents were satisfied with their operation teaching and experience. By the time they finished the STRD, only 9.68% of the residents, which was far less than the percentages generated from international surveys, thought they could perform phacoemulsification cataract surgery independently, which was the basic surgery of ophthalmology [3, 6, 7]. A possible reason could be in comparison to STRD in high-income countries, the under-going Ophthalmology STRD in Guangdong Province focuses more on the diagnosis and treatment principles of diseases, but less on the independent surgical procedures. The strained doctor-patient relationship is one of the reasons for this situation in China [13, 15]. The lack of surgical procedures in the STRD programs does not mean that they get ignored completely, especially in Guangdong Province. Under the guidance of the superior clinical tutors, every resident has the opportunity to see patients in outpatient clinics. As for teaching surgical procedures, most residents can learn through assisting operations, while few of them could perform the operations independently. In addition, wet labs and/or surgical simulators had been used for surgical training in China, especially in Zhongshan ophthalmic center, which has been proved to be effective in training ophthalmology operations [16–18]. Surgical skills training in ophthalmology is still challenging globally [17, 19]. Teaching in an operating room is complicated for both the teachers and the residents, and it is also likely to expose patients to extra risks. The integration of virtual reality surgical simulation and wet labs for clinical judgment and technical skill assessment could be encouraged, especially in China [20].

The most confusing result from the survey was that the satisfaction of cornea and orbit department training experience were the lowest. The percentages of the involved residents who viewed cornea and orbit as their aspired areas were 18.9% and 4.5%, respectively, which were much lower than the results from other countries [6]. In fact, it has been proved that if the residents were well-trained, the clinical outcome of keratoplasty done by residents were similar with those done by experienced surgeons [21]. The potential reason for the low satisfaction was that not all the training bases supported that residents could perform the keratoplasty procedures or the orbital surgeries, due to the medical environment in the real world on the aspects from the patients demands, the training plan of the hospital or the subspeciality, the social and economic efficiency. Consequently, it is time for the training director to emphasize the improvement of supervision during the cornea and orbit subspeciality training, which could lead to possible clinical growth among residents.

When asked about career preferences in this survey, the most common were cataract (67.1%), refractive surgery (42.3%), vitreoretinal (36.5%), and Optometry (28.7%), which was different from the results of oculoplastic (31.4%), vitreoretinal (25.1%), glaucoma (24.6%) and cornea (24.0%) in the UK [7]. The potential reasons for this difference are not clear but are worthy to be explored. For Ophthalmology healthcare in China, appropriate policy guidance should be in place to ensure all the ophthalmology subspecialties have enough talents to promote professional development, and to avoid the shortage of ophthalmologists, especially in areas of cornea (18.9%), ocular traumas (10.4%), pediatrics strabismus (9.8%), uveitis (5.9%), orbit (4.5%) and genetic (1.1%), where the percentage of career preference/aspiration was below than 20% [22, 23].

## Conclusions

In conclusion, the STRD program in Guangdong Province has achieved a comparable satisfaction and has been well-received by trainees. Married residents intend to be more satisfied than singles. The current curricula have significantly enhanced the clinical experiences and confidence of residents, thereby improving their competency. However, there is still room for improvement, particularly in the area of operation training. Our experience with ophthalmology STRD in Guangdong Province serves as a valuable reference for the assessment and enhancement of STRD programs throughout China.

### Electronic supplementary material

Below is the link to the electronic supplementary material.

Supplementary Material 1

## Data Availability

The anonymized dataset used and/or analyzed during the current study is available from the corresponding authors on reasonable request.

## Abbreviations

STRD: Standardized training for resident doctors
PGY: Postgraduate years
