# Supplementary material for: Standard ophthalmology residency training in China: an evaluation of resident satisfaction on training program in Guangdong Province
Source: BMC Med Educ. 2023 Aug 3;23:550. doi: 10.1186/s12909-023-04527-3 (PMC10401789; doi:10.1186/s12909-023-04527-3)
Supplement: Supplementary file 1 — Supplementary Material 1 [file 12909_2023_4527_MOESM1_ESM.docx]

**Questionnaire for Standard Ophthalmology Residency Training in Guangdong Province**

**(English Version)**

**Demographic information**

1. Gender： A. Male B. Female
2. Age： A.21-25 B.26-30 C.> 30
3. Level of training：A. postgraduate year 1 B. postgraduate year 2 C. postgraduate year 3
4. Education：A. Bachelor B. Master C. Doctor
5. Graduate university：
6. Working hospital：
7. Marital status：A. Single B. Married C. Divorced

**Working environment**

1. How many days need to spend on working?

A.< 6 days B. 6 days C.7 days D. As appropriate

1. Monthly income

A.3000-4000 Chinese yuan B.4001-5000 Chinese yuan C.5001-6000 Chinese yuan D.6001-7000 Chinese yuan E. >7000 Chinese yuan F. Month-aid for graduate

1. Is the income worthy of work?
2. Yes B. No
3. In your opinion, is your current income commensurate with your initial investment in education?
4. Income exceeds initial investment B. Income and initial investment are basically commensurate C. The income is lower than the upfront investment
5. Satisfaction score of the doctor-patient relationship in the current hospital

A.<59 B.60-80 C.80-90 D. >90

1. The number of family members practicing medicine?
2. None B. One C. More than one

**Clinical work**

1. The most time-consuming tasks as an ophthalmology resident
2. Out-patient clinic B. Surgery or operations C. Medical records D. doctor-patient communication
3. The time proportion writing medical documents spent in clinical work

A.<20% B.20-40% C.40-60% D.60-80% E. >80%

1. The proportion of additional knowledge/training on clinical knowledge
2. reading journals/books/compact disks___% B. participating in continuing education courses___% C. communicating with other residents___% D. contacting residency mentors for advice___% E. case discussion___% F. attending seminars and meetings___%
3. Other___%
4. After the first-year training, which of the following clinical examinations will you be able to complete independently? (Multiple choices)
5. Slit lamp B. Slit lamp indirect ophthalmoscope C. Direct ophthalmoscope D. Indirect ophthalmoscope E. Gonioscopy F. Applanation tonometer G. Subjective refraction
6. At the last year training, which of the following clinical examinations will you be able to complete independently? (Multiple choices)

A.Slit lamp B. Slit lamp indirect ophthalmoscope C. Direct ophthalmoscope D. Indirect ophthalmoscope E. Gonioscopy F. Applanation tonometer G. Subjective refraction H. Not available

1. After the first-year training, which of the following reports will you be able to analyze independently? (Multiple choices)
2. corneal topography B. UBM C. B scans D. OCT E. FFA F. Visual field G. Visual electrophysiology H. synoptometer test I. NONE
3. At the last year training, which of the following reports will you be able to analyze independently? (Multiple choices)

A. corneal topography B. UBM C. B scans D. OCT E. FFA F. Visual field G. Visual electrophysiology H. synoptometer test H. Not available

1. After the first-year training, which of the following operations will you be able to handle independently? (Multiple choices)
2. subconjunctival injection B. retrobulbar injection C. anterior chamber paracentesis D. intravitreal injection E. None
3. At the last year training, which of the following operations will you be able to handle independently? (Multiple choices)

A. subconjunctival injection B. retrobulbar injection C. anterior chamber paracentesis D. intravitreal injection

1. Which of the following surgery will you be able to complete? (Multiple choices)
2. chalazion excision B. sutures of the eyelid injury C. corneoscleral suture D. phacoemulsification E. None
3. Subspeciality preference (most three choices)

A. Cataract B. Glaucoma C. Cornea D. Vitreo-retina E. Medical retina F. Uveitis G. Orbit H. Ocular traumas I. Optometry J. Refractive surgery K. Paediatrics & strabismus L. Oculoplastics M. Genetic N. Other____

1. The main factors influencing the choice of subspeciality (most three choices)
2. interest B. salary C. flexibility D. low technical difficulty E. occupational accomplishment F. a wide range of employment opportunities

G. Other____

**Research**

1. The time research spent every week

A.zero B.1-5 hours C.5-10 hours D. >10 hours

1. Types of research participation
2. Basic research B. Clinical research C. Both D. Neither
3. The number of academic articles published on Chinese and international medical journal

Chinese articles ，English articles

1. The reasons for publishing articles or attending conferences

A. an interesting pursuit B. solving clinical problems C. career promotion pressure D. enhance their academic status E. helping to obtain medical degrees

1. The influence of participating in academic activities during clinical work

A. leading to fatigue and being a waste of time B. be beneficial in enhancing clinical thinking C. None

1. Frequency of attending academic meetings
2. zero B. 1 or 2 times per year C. 3 to 5 times per year D. > 5 times per year

**General Satisfaction**

1. The overall satisfaction of this training program

A. Satisfied B. Neutral C. Dissatisfied

1. How do you feel about the quality of teaching in the following settings?

|  | Satisfied | Neutral | Dissatisfied |
| --- | --- | --- | --- |
| Pre-employment training |  |  |  |
| Clinic/outpatient office Hospital-based rounds |  |  |  |
| Operating teaching |  |  |  |
| Case discussion |  |  |  |
| Teaching grand rounds |  |  |  |
| Hospital-based academic activities |  |  |  |
| Academic conferences |  |  |  |

1. How do you feel about the operative experience in the following areas?

|  | Satisfied | Neutral | Dissatisfied |
| --- | --- | --- | --- |
| Case variety |  |  |  |
| Case complexity |  |  |  |
| Case volume |  |  |  |
| Cataract |  |  |  |
| Glaucoma |  |  |  |
| Cornea |  |  |  |
| Retina |  |  |  |
| Strabismus |  |  |  |
| Orbital |  |  |  |

1. Is the program helpful for the future clinical work?

A. Satisfied B. Neutral C. Dissatisfied

**广东省眼科住院医生规范化培训调查问卷**

**基本信息**

1. 性别： A.男 B.女
2. 年龄： A.21-25 B.26-30 C.30以上
3. 规培年级：A.第一年 B.第二年 C.第三年
4. 学历： A.本科 B.硕士 C.博士
5. 毕业学校：
6. 现在所在医院：
7. 婚姻及生育状况： A.未婚 B.已婚 C.离异

**工作环境**

1. 每周工作天数（包括临床工作与科研）：

A.< 6 天 B.6天 C.7天 D.视情况而定

1. 每月收入：

A.3000-4000元 B.4001-5000元 C.5001-6000元 D.6001-7000元 E.7000元以上

1. 在你看来，你现在所得工资和现工作量应得工资相称吗？

A.收入超过应得工资 B.收入和应得工资基本相称 C.收入低于工作应得

1. 在你看来，你现在的收入和前期学习投入相称吗？

A.收入超过前期投入 B.收入和前期投入基本相称 C.收入低于前期投入

1. 你如何评价目前所在医院的医患关系的满意度？

A.<59分 B.60-80分 C.80-90分 D.90分以上

1. 是否有家庭成员从医？

A.无 B.有1位 C.有，多于1位

**临床**

1. 在临床工作中，占比例最多的工作是：

A.门诊 B.手术 C.处理文书工作 D. 与患者及家属沟通

1. 在临床工作中，医疗文书书写占时间比例是：

A.<20% B.20-40% C.40-60% D.60-80% E. 80%以上

1. 你的临床知识的来源比例是：

A.自学课本 % B.继续医学教育讲课 % C.同年资医生间交流讨论 %

D.上级医生查房讲解 % E.科室病例讨论 % F.医院学术会议 %

G.其他，请注明（ ） %

1. 第一年轮转结束后，你能独立完成以下哪些临床检查（多选）：

A.裂隙灯 B.前置镜 C.直接检眼镜 D.间接检眼镜 E.房角镜 F.压平眼压计检查

G.主觉验光

1. 轮转最后一年，你能独立完成以下哪些临床检查（多选）：

A.裂隙灯 B.前置镜 C.直接检眼镜 D.间接检眼镜 E.房角镜 F.压平眼压计检查

G.主觉验光 H.未到轮转最后一年

1. 第一年轮转结束后，能独立分析以下哪些检查报告（多选）：

A.角膜地形图 B.UBM C.B超 D.OCT E.眼底荧光造影 F.视野检查

G.电生理检查 H.同视机 I.以上都不会

1. 轮转最后一年，能独立分析以下哪些检查报告（多选）：

A.角膜地形图 B.UBM C.B超 D.OCT E.眼底荧光造影 F.视野检查

G.电生理检查 H.同视机 I.未到轮转最后一年

1. 第一年轮转结束后，能独立完成以下哪些操作（多选）：

A.结膜下注射 B.球后注射 C.前房穿刺 D.玻璃体腔注药 E.以上都不会

1. 轮转最后一年，能独立完成以下哪些操作（多选）：

A.结膜下注射 B.球后注射 C.前房穿刺 D.玻璃体腔注药

1. 完成以下哪些手术操作（多选）：

A.睑板腺囊肿切开 B.眼睑缝合 C.角巩膜缝合 D.白内障超声乳化 E.以上都没有

1. 你更愿意成为以下哪个专科的眼科医生（可选3个）：

A.白内障 B.青光眼 C.角膜 D.眼底外 E.眼底内 F.葡萄膜炎 G.眼眶眼肿瘤

H.眼外伤 I.屈光 J.准分子激光 K.斜弱视 L.整形 M.遗传病 N.其他：

1. 你选择专科的主要影响因素是（可选3个）：

A.兴趣 B.收入高 C.工作时间自由 D.工作难度低 E.有成就感 F.就业机会多

G.其他：

**科研**

1. 每周参加科研时长：

A.0小时 B.1-5小时 C.5-10小时 D.10小时以上

1. 你参与科研的类型是：

A.基础研究 B.临床研究 C.二者都有 D.均未参加

1. 目前在学术杂志上发表文章篇数是：

中文杂志 篇，英文杂志 篇

1. 参与科研的主要目的

A.兴趣 B.解决临床问题 C.职称晋升 D.提高学术地位 E.学位要求

1. 认为参加科研对临床工作产生了什么影响？

A.使临床工作精力时间不足 B.促进了临床思维 C. 没有影响

1. 每年参与的学术交流活动次数为：

A.0次 B.1-2次 C.3-5次 D.5次以上

**满意度**

1. 你对于眼科住院医师规范化培训（以下统称“规培”）的总体满意度为

A.满意 B.中立 C.不满意

1. 对于规培期间的**教学培训**的满意度（请在表格中对应选项打“√”）：

|  | 满意 | 中立 | 不满意 |
| --- | --- | --- | --- |
| 岗前培训 |  |  |  |
| 门诊教学 |  |  |  |
| 手术教学 |  |  |  |
| 病例讨论 |  |  |  |
| 教授教学查房 |  |  |  |
| 院内学术活动 |  |  |  |
| 学术会议 |  |  |  |

1. 对于规培期间的**手术培训**的满意度（请在表格中对应选项打“√”）：

|  | 满意 | 中立 | 不满意 |
| --- | --- | --- | --- |
| 病例多样性 |  |  |  |
| 病例复杂程度 |  |  |  |
| 手术总量 |  |  |  |
| **白内障**手术 |  |  |  |
| **青光眼**手术 |  |  |  |
| **角膜**手术 |  |  |  |
| **眼底**手术 |  |  |  |
| **斜视**手术 |  |  |  |
| **眼眶**手术 |  |  |  |

1. 你是否觉得规培期间的培训帮助你为未来临床工作做好准备？

A.同意 B.中立 C.不同意
